# Case of Injury of the Head, Attended with Compression from Effusion of Blood beneath the Dura Mater, in a Boy Whose Cranium Afforded an Example of Defective Ossification to a Great Extent

**Published:** 1827-02

**Authors:** 

**Affiliations:** Surgeon to the Middlesex Infirmary, &c.


*Case of Injury of the Head, attended with Compression from Effusion of Blood beneath the Dura Mater, in a Boy whose Cranium afforded an example of defective Ossification to a great extent.* By Mr. Boyle, Surgeon to the Middlesex Infirmary, &c.

On the morning of the 21st of September, I was called upon to visit Henry Slark, a boy of nine years old, residing in George-street, Grosvenor-square, he having been thrown upon the pavement from a horse, and it being supposed that he laboured under an extensive fracture of the skull. On my arrival, I was informed that the boy's head (to use the words of his friends,) had never closed in front, and that his parents were frequently alarmed by his falling down in the streets without any apparent cause.

On examination of the head, it was found to be of a hydrocephalic form. A large open space was pointed out, commencing at the situation of the anterior fontanel, and widening as it ascended on the right side, in the course of the sagittal suture.

This was stated to have existed from birth; but all the other parts of the head were supposed to have been perfect, except in form, previously to the accident.

The symptoms, which were a total suspension of the mental faculties, stertorous breathing, dilatation of the pupils, with insensibility to light, a low, rapid, and occasionally irregular pulse, and coldness of the extremities, denoting serious mischief. A careful examination of the head was resorted to, and marks of external violence were detected behind the posterior superior angle of the right parietal bone. On pressure being made here with the fingers, a depression to some extent was felt. The surrounding integuments were now (three hours after the accident) greatly tumified; and, as nothing but irregularly projecting boundaries of the injured portion could be distinguished, it was believed by myself and the other gentlemen present, amongst whom were Mr. Jewel and Mr. Love, that broken fragments of bone were driven between the surrounding sound or unhurt parts of the skull and the dura mater. This opinion, however, did not at all regulate the course of treatment adopted. Blood had been taken from the arm, and active purgatives had been administered, without any improvement, but, on the contrary, an increase of the threatening symptoms: it was, therefore, considered expedient, at all events, to divide the pericranium over the apparent seat of the injury, for the purpose of ascertaining and removing, if possible, the immediate cause of mischief. This I accordingly did by two free crucial incisions. The scalp was reflected back; the dura mater was exposed; the action of the brain was seen and felt, but no broken bone was to be found; this part of the dura mater having been originally unprotected, and uncovered by any thing but the scalp. Effused blood, however, (unquestionably the cause of the symptoms,) was found lying on the dura mater, and removed. The distended vessels, now divided, bled freely, and it was necessary to secure three arterial branches.

The parts having been brought together and dressed, the boy gave direct answers to two or three questions, and the pupils evinced a slight degree of irritability on the approach of a lighted candle. The pulse, however, was now extremely weak, hurried, and irregular; and no alvine evacuation had as yet been obtained. Twelve leeches were ordered to be applied to the affected side of the head, followed by cooling lotions; three grains of calomel, and an equal quantity of extract of colocynth, were given; and a purgative enema, containing an ounce of spirits of turpentine, was directed to be administered every two hours, till free and frequent evacuations should be produced.

On the following morning, the boy was irritable and restless, but sensible on interrogation; and the pupils, which were now about their natural size, contracted on the approach of light; the bowels had been freely opened. There was still, however, a good deal of stupor; the pulse was 116, and small; the skin was hot, and there was much thirst. A blister was ordered to the nape of the neck, the saline mixture to be administered, and the strictest attention to the antiphlogistic treatment was enjoined.

From this time there was a gradual improvement, till the 25th, when the boy became extremely restless and feverish, and complained of severe pain in the forehead. Three leeches were applied to the seat of pain, and it was found necessary to repeat them in the course of the day. As no regular sleep had been obtained since the time of the accident, an anodyne draught was administered. These measures were productive of relief and several hours' sound sleep.

No unfavourable change afterwards took place, and on the 1st of October the wound from the operation had quite healed, and the little patient was free from all complaint but debility.

It is worthy of observation that, on the subsidence of the swelling and tension of the scalp, attendant on the violence which it sustained, a vacuity, from a deficiency of the parietal bones, but more particularly the right, was ascertained to exist in the direction of the sagittal suture; being at its narrowest part about two, and at its broadest about four, inches wide. A small tooth-like process secured the posterior superior angle of the right parietal to its fellow, and to the occipital. Posteriorly and inferiorly to this was the more immediate seat of the mischief arising from the accident. Here also an original defect was observed in the ossific process, to the extent of about three inches at its narrowest, and about four and a half at its broadest part; the natural situation of the lamdoidal suture being near its centre. The head, from the nasal process of the frontal bone to the occipital protuberance, measured seventeen inches and three-quarters, whilst its breadth, from the tip of one ear to that of the other, was only eleven inches and three-quarters; affording altogether a curious specimen of the occasional irregularities of nature in the performance of her operations.

*4, Cleveland-square, St. James's; Oct. 3d,* 1826.

---

